# Morphological, molecular, and histopathological studies on *Hadjelia truncata* from *Columba livia domestica* and its role as an environmental biological indicator

**DOI:** 10.2478/jofnem-2023-0050

**Published:** 2023-11-16

**Authors:** Mohammed Albeshr, Rajwa Alsharief, Fatimah Al-Otibi, Esam M. Al-Shaebi, Osama B. Mohammed, Rewaida Abdel-Gaber

**Affiliations:** Department of Zoology, College of Science, King Saud University, Riyadh, Saudi Arabia; Department of Botany and Microbiology, College of Science, King Saud University, Riyadh, Saudi Arabia

**Keywords:** *Columba livia*, Parasites, Identification tools, Negative impacts, Biomonitoring

## Abstract

Pigeons are a cosmopolitan group of birds with abundant and large populations associated with human activities. This study focused on determining parasitic infections within domestic pigeons (*Columba livia domestica*). Forty-eight pigeons were examined for infections, of which 29.16% were infected with a nematode parasite, identified as *Hadjelia truncata* (Habronematidae), under the koilin layer of their gizzards. The population of nematodes in infected gizzards did not exceed 20 adult worms. DNA from the gizzard worms was extracted and subjected to PCR using primers that amplify the partial 18S rDNA and cytochrome C oxidase subunit I (COX I) regions. Identification of this parasite based on microscopic study revealed the presence of trilobed lips with cephalic papillae and amphidial pores, as well as other characteristic features. In males, spicules were unequal with the presence of six pedunculated pairs of caudal papillae (4 pre- and 2 post-anal) and a tail surrounded with caudal ala. In females, the vulva was a rounded aperture located in front of the posterior end of the esophagus and uteri, which was filled with numerous embryonated eggs. DNA Sequences from partial 18S rDNA were homologous to sequences obtained from *H. truncata* in GenBank with a high percentage of identity. DNA sequences from mitochondrial gene COX I, however, were unique, and they were the first sequenced for *H. truncata*, since no sequences for this taxon were previously available in GenBank. Histopathological examination revealed enlargement of infected gizzards in comparison to non-infected ones, with the presence of necrosis and interstitial infiltration in the koilin layer. Concentrations of heavy metals (Fe, Cu, Zn, Cd, Cr, and Co) were measured using inductivity-coupled plasma in tissues (liver, muscles, and gizzards) from infected and non-infected pigeons as well as their parasites. Results showed different affinities of metals to tissues. Recovered parasites can minimize element concentration from their pigeon tissues. In Saudi Arabia, this study was considered the first report identifying pigeon nematodes and evaluating of the effects of their pathogenicity on the animals’ welfare, as well as their application as a useful tool for monitoring environmental pollution.

## Introduction

Domestic pigeons (*Columba livia domestica*) are common free-living birds belonging to the order Columbiformes coexisting in the environment with humans (Al-Barwari and Saeed, 2012; Alkharigy et al., 2018). Pigeons serve as reservoir hosts for a variety of pathogens that can be transmitted to other bird species as well as humans (Sari et al., 2008; Hamzah et al., 2020; Salem et al., 2021). Nematodes are helminth species that can have a direct or indirect life cycle. *Hadjelia* species (Habronematidae, Spiruridea) are widespread nematode species in pigeons (Abd-Alhadi and Al-Awady, 2020). The adult parasite settles in the gizzards of birds, including pigeons, and its eggs are released with the birds’ droppings. It has an indirect lifecycle, and some beetles (i.e., *Alphitobius diaperinus*, within the family Tenebrionidae) have been reported to serve as intermediate hosts in their hemocoel, in which the infectious stage (L3) develops, similar to those of the superfamily *Spiruroidea* (Alborzi and Rahbar, 2012).

Diagnosis is currently determined by the presence of nematodes under the koilin layer of the gizzard during necropsy. *H. truncata* Creplin, 1825 has been reported as a *Hadjelia* species widely distributed in commercial pigeons (Tadros and Iskander, 1975; Al-Attar and Abdul-Aziz, 1985; Farah, 1988; Appleby et al., 1995; Razmi et al., 2007; Junker and Boomker, 2007; Shubber, 2010; Sentíes-Cué et al., 2011; Alborzi and Rahbar, 2012; Nabavi et al., 2013; Al-Moussawi and Jassim, 2015; Oryan et al., 2016; Jameel et al., 2016; Khordadmehr et al., 2016, 2018; Abd-Alhadi and Al-Awady, 2020). Infection with these nematodes generates various pathological conditions such as impaired growth, poor feed consumption, lowered production, and decreased performance (Bizhga et al., 2011). Moreover, there is a possibility of transmission of gizzard worms between domestic and wild birds that share the same habitats. All of this can cause significant economic losses (Kelly et al., 2013).

The basic discriminating tool for the *Hadjelia* species was based on morphological examination of the male worms, with special reference to the cloacal papillae at the posterior end of the body (Razmi et al., 2007; Shubber, 2010; Al-Moussawi, 2015; Jameel et al., 2016; Khordadmehr et al., 2018). Extensive studies have been conducted to examine various aspects of parasite-host interactions between habronematid nematodes, such as host immune responses and effects on host behavior and health (Sentíes-Cué et al., 2011; Nabavi et al., 2013; Oryan et al., 2016; Abd-Alhadi and Al-Awady, 2020). Recently, molecular tools have been the most efficient and accurate means to detect many species of Spirurida and to screen genetic variation among populations. The nuclear small-subunit ribosomal DNA (18S rDNA) (Al Quraishy et al., 2020) and the mitochondrial cytochrome C oxidase subunit 1 (COX I) (Binkiené et al., 2021) were used for phylogenetic identification and discrimination between different nematode species.

This study was designed to shed more light on habronematid parasites, especially *Hadjelia* species, that infect domestic pigeons. It accomplishes this using morphological and molecular identification tools, as well as observations of the histopathological effects of these parasites on their hosts.

## Materials and Methods

### Sample collection

A total of 48 domestic pigeons, *Columba livia domestica* (family: Columbidae), were collected from different areas in Riyadh (Saudi Arabia), from September 2022 to June 2023. Pigeons were transferred to the lab of parasitology for further examination. All pigeon handling procedures followed the institution’s accepted guidelines on the care and use of animals in research.

### Isolation and fixation of parasites

Pigeons were autopsied after being euthanized with lethal chloroform, and then alimentary tracts were removed to be examined under a stereomicroscope (Nikon SMZ18, NIS ELEMENTS software). The esophagus, crop, proventriculus, gizzard, duodenum, jejunum, ileum, ceca, and rectum were separated and placed into Petri dishes. Longitudinal incisions were made in all parts to expose their contents, and then nematode parasites were gently collected using fine-tip forceps in separate Petri dishes with normal saline (0.9%) and then fixed in either 70% ethanol (for light microscopic study) or 95% ethanol (for molecular study), or stored in −20°C (for heavy metal detection). Parasitological terms of the prevalence and mean intensity were calculated using equations per Bush *et al*. (1997).

### Light microscopic (LM) study

Parasites were cleared in lactophenol and then different body parts were photographed with the aid of a Leica DM 2500 microscope (NIS ELEMENTS software, ver. 3.8). Identification of the parasites was done according to Yamaguti (1961). Measurements were taken using ImageJ 1.53e software (Wayne Rasband and contributors, National Institute of Health, USA). Dimensions were given in micrometers (µm) and expressed as a range followed by mean in parentheses.

### Molecular analysis

Genomic DNA (gDNA) was extracted using QIAamp® DNA Mini Kit (Qiagen, Germany) from ethanol-preserved samples with consideration of protocol steps. A partial fragment of the 18S rDNA region was amplified by PCR using the primer pair Nem 18SF, 5′-CGC GAA TRG CTC ATT ACA ACA GC-3′, and Nem 18SR, 5′-GGG CGG TAT CTG ATC GCC-3′, following the conditions described by Floyd et al. (2005). A partial fragment of the COX I gene was amplified using LCO1490, 5′-GGT CAA CAA ATC ATA AAG ATA TTG G-3′, and HC02198, 5′-TAA ACT TCA GGG TGA CCA AAA AAT CA-3′, with the recommended conditions of Folmer et al. (1994).

The PCR products were verified on a 1.5% agarose gel (Sigma-Aldrich, Missouri, USA) in 1 × Tris-acetate–EDTA (TAE) and post-stained with SYBR Safe DNA gel dye (Thermo Fischer Scientific, Ottawa, Canada) against the GeneRuler 100 bp Plus ready-to-use DNA ladder as a molecular weight marker (Fermentas, Lithuania) (Catalano et al., 2010). They were then visualized using a gel documentation system (Image Analyzer, Malvern, UK). PCR products were sent to Macrogen (Seoul, South Korea) to sequence the DNA. To identify related sequences, a BLAST search was conducted on the NCBI database. The DNA sequences were aligned using multiple alignments of CLUSTAL-X software (Thompson et al. 1997). Phylogenetic trees were constructed with maximum parsimony using MEGA ver. 7.0 (Kumar et al., 2018). Bootstrap analysis was performed based on 1,000 replicates to assess the robustness of the tree topologies.

### Histopathological study

Tissue specimens from the gizzards of pigeon-containing parasites were processed by paraffin embedding technique after fixation in 10% neutral buffered formalin for 24 hr according to the method of Banchroft and Stevens (1982). Specimens were washed in tap water and dehydrated in serial dilutions of ethyl alcohol. They were then cleared in xylene and embedded in paraffin. A cross-section of the gizzard was made 5 µm thick using a microtome, followed by deparaffinization, staining with hematoxylin, and eosin (H&E) stain examination. Sections were examined and photographed using a Leica DM 2500 microscope (NIS ELEMENTS software, version 3.8).

### Heavy metal detection

Pigeon tissues (liver, muscle, and gizzard) and parasites were analyzed to detect heavy metals according to the procedure described by UNEP/FAO/IOC/IAEA (1984). The samples were dried at 105°C for 1 hr in an oven. Samples were digested with 2 ml of concentrated hydrochloric acid overnight. After complete digestion, the samples were diluted with distilled water and then analyzed for trace elements using inductivity-coupled plasma iCAP-6500 Duo (Thermo Scientific, UK). Values of all monitored heavy metals are presented in mg/g wet weight. According to Sures et al. (1999), the bioaccumulation factor (BF) was determined as a ratio of the metal concentration in the parasite to that in the host tissue. Values from chemical analyses were presented as mean ± SE. Data obtained were analyzed using the SPSS v.18 software package (SPSS, Chicago, Illinois, USA). *P-*value ≤ 0.05 was considered statistically significant.

## Results

Fourteen specimens of the examined domestic pigeons out of 48 (29.16%) were found to be naturally infected with *Hadjelia runcate* Creplin, 1825. The infection was reported under the koilin layer of the gizzard of the pigeons. No parasitized pigeon had an infection intensity that exceeded 20, with the mean intensity being 14.31.

### Morphological examination

Adult worms are elongated with slender bodies, with the cuticle striated transversally and lateral alae absent. The mouth is surrounded by two well-developed trilobed lips. Interlabia are present. Cephalic papillae and amphidial pores are present on the outer surface of the lips. The pharynx is tubular. The oesophagus is divided into an anterior muscular portion and a glandular portion.

### Male worms

**Figure 1:**
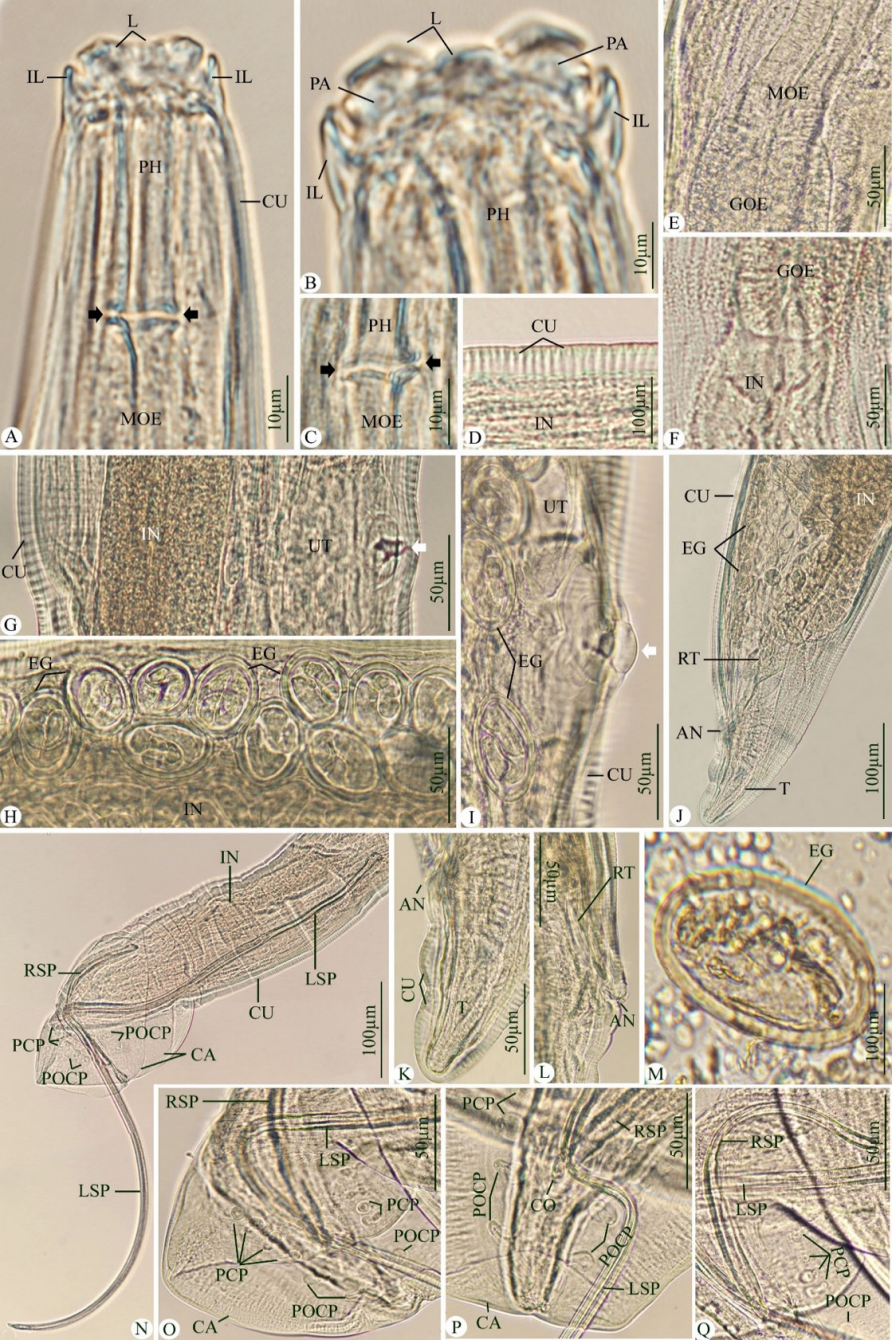
Photomicrographs for the *Hadjelia truncata* infecting the domestic pigeons. (A–F) Anterior extremity. (G–I) Middle part for female worms. (J–L) The posterior extremity of females. (M) Embryonated egg in feces. (N–Q) The posterior extremity of male worms. Note: L, lips; IL, interlabia, PA, papillae; CU, cuticle; PH, pharynx; MOE, muscular oesophagus; IN, intestine; GOE, glandular esophagus; Black arrow, constriction at the end of pharynx; EG, embryonated eggs; RT, rectum; IN, intestine; T, tail; AN, anal opening; White arrow, vaginal opening, UT, uterus; RSP, right spicules; LSP, left spicules; PCP, pre-cloacal papillae; POCP, post-cloacal papillae; CA, caudal alae; CO, cloacal opening.

(Figure 1, Table 1): The body is 6.201–8.023 (7.270) mm long and 0.191–0.219 (0.204) mm wide. The pharynx is 0.036–0.041 (0.038) mm long and 0.074–0.093 (0.085) mm wide. The muscular oesophagus is 0.153–0.176 (0.167) mm long and the glandular portion ranges from 1.599–1.758 (1.623) mm long. The nerve ring is located at 0.184–0.201 (0.197) mm from the anterior end of the body. Testes are coiled and reflexed, extending through four-fifths of the body. The posterior extremity is provided with six pedunculated pairs of caudal papillae (4 preanal and 2 postanal). Two unequal spicules are present, the left one measuring 1.332–1.511 (1.416) mm long, and the right one 0.270–0.320 (0.284) mm long. Gubernaculum is absent. The tail is slightly twisted and bent in the ventral direction, carrying a well-developed caudal ala on each side.

**Table 1.** Morphological characteristics of male worms for *Hadjelia truncata* from the domestic pigeons

**Source of *Hadjelia truncata***	**Body**		**Esophagus**		**Distance of nerve ring from anterior end**	**Spicule length**		**Caudal papillae**	**Tail length**
		
	**Length**	**Width**	**Muscular length**	**Glandular length**		**Right**	**Left**		
Cram, 1927	5–7	–	–	–	–	0.220	1.6	Six pairs (4 pre- & 2 post-) of caudal papillae	–
Baer, 1954	8	0.285	0.033	–	–	0.225–0.300	1.320–1.600		–
Ibrahim et al., 1995	4–8 (6.4)	–	1.8–2.7 (2.2)		–	–	–		–
Junker & Boomker, 2007	7–8	0.145–0.160	0.042–0.044	1.750–1.927	0.208–0.212	0.254–0.271	1.346–1.434		–
Razmi et al., 2007	7–9	–	–	–	–	0.34	1.26		–
Sentíes-Cué et al., 2011	6.5–9	–	–	–	–	0.35	1.27		–
Naem et al., 2013	6.5–9	0.151	–	–	–	0.35	1.27		–
Nabavi et al., 2013	1–2	–	–	–	–	0.320–0.350	1.410–1.470		–
Al-Moussawi, 2015	7–8 (7.5)	0.204–0.258 (0.231)	0.374–0.386 (0.380)	1.620–1.680 (1.650)	0.198–0.211 (0.204)	0.240–0.270 (0.255)	1.512–1.620 (1.566)		0.127–0.138 (0.132)
Oryan et al., 2016	7.5	–	–	–	–	–	–		–
Jameel et al., 2016	6.5–9	–	–	–	–	–	–		–
Khordadmehr et al., 2018	7–11	–	–	–	–	–	–		–
Present study, 2023	6.201–8.023 (7.270)	0.191–0.219 (0.204)	0.353–0.376 (0.367)	1.599–1.758 (1.623)	0.184–0.201 (0.197)	0.270–0.320 (0.284)	1.332–1.511 (1.416)		0.118–0.127 (0.122

### 2- Female worms

(Figure 1, Table 2): The body is 12.270–18.583 (17.792) mm long and 0.256–0.314 (0.287) mm wide. The pharynx is 0.041–0.044 (0.042) mm long and 0.099–0.012 (0.11) mm wide. The muscular oesophagus is 3.515–3.759 (3.601) mm long and its glandular portion is 2.160–2.211 (2.181) mm long. The nerve ring is located at 0.253–0.295 (0.282) mm from the anterior end of the body. The vulva is located in front of the posterior end of the oesophagus at a distance of 3.011–3.054 (3.027) mm from the anterior extremity of the body, and appears as a rounded aperture with a thickened cuticular edge. The vagina is elongated and divided into two divergent branches of the uteri that are filled with numerous oval and thick-shelled eggs, which become embryonated *in utero.* Eggs are 0.046–0.051 (0.048) mm long and 0.029–0.033 (0.031) mm wide. The tail is short with a bluntly rounded tip.

**Table 2.** Morphological characteristics of female worms for *Hadjelia truncata* from the domestic pigeons

**Source of *Hadjelia truncata***	**Body**		**Esophagus**		**Distance from anterior end**		**Eggs**			**Tail length**
			
	**Length**	**Width**	**Muscular length**	**Glandular length**	**Nerve ring**	**Vulva opening**	**Length**	**Width**	**Status**	
Cram, 1927	10–16	0.300	–	–	–	2.6–16	0.027	–	Numerous embryonated eggs covered with thick-shelled	–
Baer, 1954	8–16	0.143–0.285	–	–	–	1.640–3.000	–	–		–
Ibrahim et al., 1995	17–22 (20)	–	2.0–2.4 (2.4)		–	1.5–2.5 (2)	0.050	0.030		–
Junker & Boomker, 2007	10–11	0.140–0.217	0.005–0.007	1.948–2.076	0.159–0.185	1.691–2.238	0.050–0.053	0.032–0.035		0.121–0.138
Razmi et al., 2007	13–17	–	–	–	–	–	–	–		–
Al-Moussawi, 2008	14–15.22 (14.62)	0.168–0.199 (0.183)	0.512–0.57 (0.54)	0.589–0.622 (0.605)	0.003–0.024 (0.015)	–	–	–		0.113–0.150 (0.132)
Mohammad & Al-Moussawi, 2011	11.10–14.88 (13.15)	0.18–0.28 (0.23)	0.450–0.566 (0.461)	1.47–3.32 (2.435)	–	1.575–2.730 (2.281)	0.033–0.057 (0.044)	0.015–0.041 (0.034)		–
Sentíes-Cué et al., 2011	12–16.5	–	–	–	–	–	–	–		–
Naem et al., 2013	12	16.5	0.229	–	–	2.155	–	–		–
Nabavi et al., 2013	3–5	–	–	–	–		0.054–0.059	0.030–0.032		
Al-Moussawi & Jassim, 2015	23.226–26.156 (24.594)	0.231–0.312 (0.2782)	3.510–3.666 (3.588)	–	0.260–0.312 (0.291)	3.276–3.413 (3.364)	0.052–0.104 (0.078)	0.021–0.312 (0.026)		0.104–0.234 (0.148)
Al-Moussawi, 2015	8–17 (12)	0.147–0.270 (0.224)	2.750–3.648 (3.379)	2.002–2.835 (2.215)	0.178–0.206 (0.192)	1.501–3.022 (2.108)	0.046–0.048 (0.047)	0.024–0.0408 (0.047)		0.145–0.230 (0.196)
Oryan et al., 2016	19.8	–	–	–	–	–	0.045	0.031		–
Jameel et al., 2016	12–16.5	–	–	–	–	–	–	–		–
Khordadmehr et al., 2018	15–20	–	–	–	–	–	0.043–0.045	0.020–0.030		–
Present study, 2023	12.270–18.583 (17.792)	0.256–0.314 (0.287)	3.515–3.759 (3.601)	2.160–2.211 (2.181)	0.198–0.225 (0.211)	3.011–3.054 (3.027)	0.046–0.051 (0.048)	0.029–0.033 (0.031)		0.118–0.132 (0.124)

### Molecular analysis

**Figure 2:**
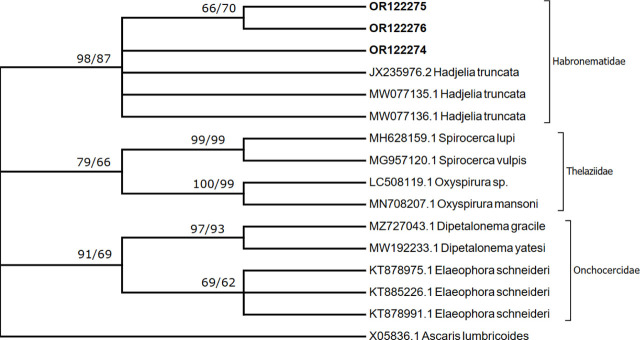
A consensus phylogenetic tree constructed with neighbor joining (NJ) and maximum likelihood (ML) methods, showing phylogenetic relationships between *Hadjelia truncata* and related taxa in NCBI GenBank with *Ascaris lumbricoides* as an outgroup. The ML and NJ trees are inferred from the 18S rDNA sequences data generated from the *H. truncata* recovered from *Columba livia domestica* (OR122274 to OR122276 given in bold) and related taxa from GenBank. Numbers indicated at branch nodes are bootstrap values (ML/NJ). Only bootstraps > 60% are shown.

**Figure 3:**
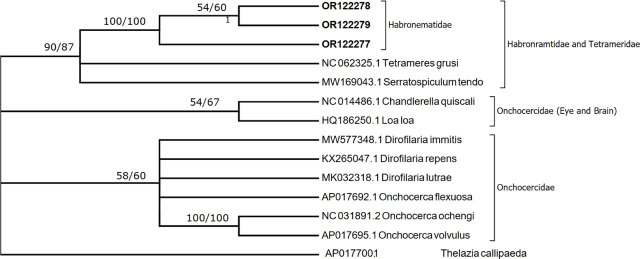
A consensus phylogenetic tree constructed with neighbor joining (NJ) and maximum likelihood (ML) methods, showing phylogenetic relationships between *Hadjelia truncata* and related taxa in NCBI GenBank. The ML and NJ trees are inferred from the Cytochrome subunit C oxidase I (COX I) DNA sequences data generated from the *H. truncata* recovered from *Columba livia domestica* (OR122277 to OR122279 given in bold) and related taxa from GenBank. Numbers indicated at branch nodes are bootstrap values (ML/NJ). Only bootstraps > 70% are shown.

(Figures 2, 3) Amplification of both partial 18S rDNA and cytochrome oxidase C subunit 1 (COX I), of the nematode *Hadjelia truncuata*, successfully yielded 900 bp and 651 bp, respectively. Three sequences of the region 18S rDNA were obtained and deposited in GenBank and given the accession numbers OR122274 to OR122276. The three sequences obtained from the nematode (*H. truncata*) during this study were identical except for one mutation (transition A to G) found on both OR122275 and OR122276 at position 116 of the alignment.

Only three sequences of the 18S rDNA related to *H. truncata* were previously available in GenBank (MW077135, MW077136, and JX235976). The first two sequences had been obtained from *H. truncata* in Iraq, while the third sequence was obtained from the USA. The sequence designated MW077136 was variable on 14 sites, including position 116 of the alignment. The sequence JX235976 was identical to the sequence OR122274 obtained in this study. Phylogenetic analysis revealed that sequences obtained in the present study group, with the only three sequences available in GenBank related to *H. truncata,* form a group (Habronematidae) with a strong bootstrap value (Figure 2). The clade designating Habronematidae was distinct from Onchocercidae and Thelaziidae (Figure 2).

Three sequences were also obtained from the COX I region and were given the accession numbers OR122277 to OR122297 in GenBank. There are no COX I sequences related to *H. truncata* available in GenBank to compare with. However, the phylogenetic tree resulting from COX I analysis revealed that sequences related to *H. truncata* are distinct from the related Tetrameridae, which were represented by *Tetrameters grusi* and *Serratospiculum tendo* (Figure 3). The three sequences of *H. truncata* obtained in the present study were variable on seven sites and showed three different haplotypes. The amino acids that resulted from the translation of the DNA sequences obtained were identical except for a site on the haplotype (OR122277) that showed serine (S), when the other two haplotypes had shown phenylalanine (F).

### Histopathological study

**Figure 4:**
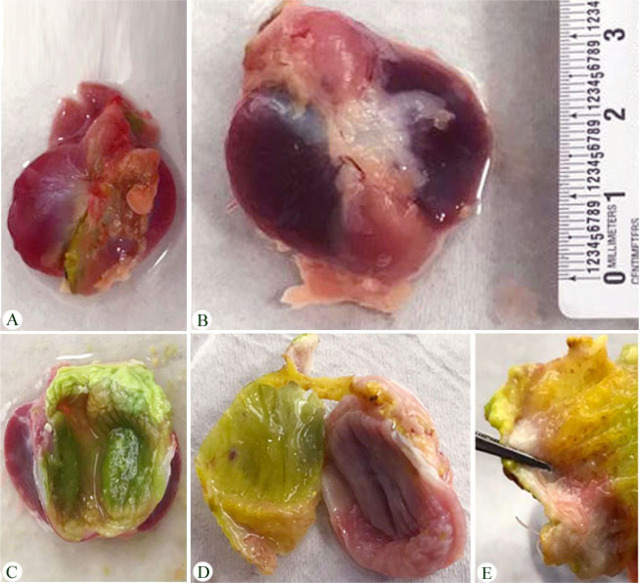
Photographs of the gizzards of the domestic pigeons. (A) Non-infected gizzard. (B) Enlargement of the infected gizzard. (C) Normal koilin layer. (D and E) Nematodes infection under the koilin layer of the gizzard.

**Figure 5:**
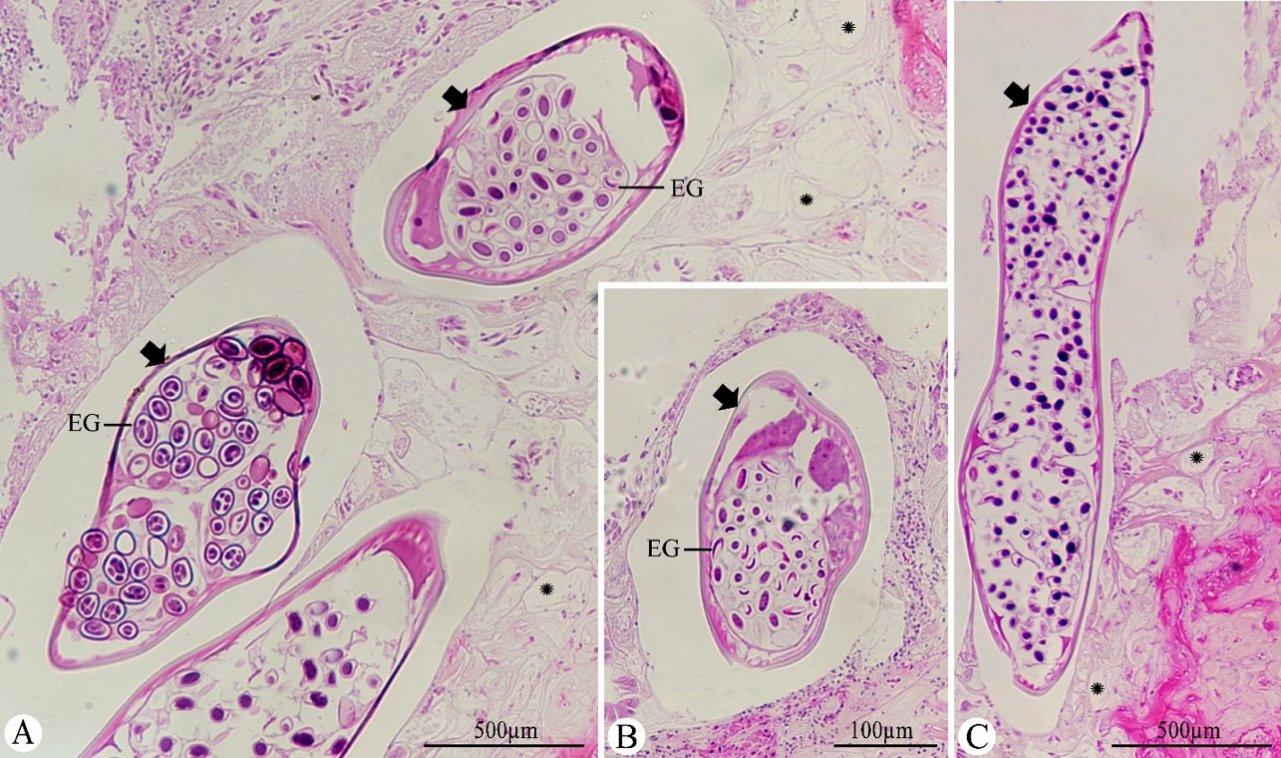
(A–C) Histopathological sections from the gizzard’s wall infected with *Hadjelia truncata*, exhibiting the transverse sections of the nematodes (black arrows) with a large number of embryonated eggs (EG). The koilin layer is disrupted and fragmented with multiple clear spaces (asterisks).

Macroscopic examination showed enlargement of the infected gizzard in the domestic pigeon (Figure 4). Moreover, there is noticeable damage to the koilin layer of the gizzard, with necrosis of the mucosal cells and interstitial infiltration of inflammatory cells in the lamina propria and muscular layer, and the presence of numerous cross-sections of nematode parasites between the koilin layer and glandular layers (Figure 5).

### Heavy metal analysis

Significant differences in concentrations of analyzed metals were observed among the different pigeon tissues (Tables 3–5). These metals were classified as essential metals of iron (Fe), copper (Cu), and zinc (Zn) and non-essential metals of cadmium (Cd), chromium (Cr), and cobalt (Co). There was a significant decrease in the concentration of metals in the pigeon tissues infected by nematode parasites compared to non-infected ones. The liver was considered as the main site of heavy metal storage, especially for Fe, while the gizzard had the lowest levels of analyzed metals.

**Table 3.** Heavy metals in the liver of the domestic pigeons and its parasite

**Heavy metals**	**Non-infected pigeon**	**Infected pigeon**	**Parasite**
Fe	5.907 ± 0.02	1.022 ± 0.02 ^a^	1.463 ± 0.02 ^ab^
Cu	0.608 ± 0.02	0.132 ± 0.01 ^a^	0.313 ± 0.00 ^ab^
Zn	0.935 ± 0.02	0.253 ± 0.02 ^a^	0.522 ± 0.02 ^ab^
Cd	0.046 ± 0.001	0.011 ± 0.001 ^a^	0.036 ± 0.001 ^ab^
Cr	0.617 ± 0.02	0.341 ± 0.01 ^a^	0.518 ± 0.02 ^ab^
Co	0.212 ± 0.01	0.104 ± 0.01 ^a^	0.114 ± 0.01 ^ab^

Values are means ±SD.

a significance at p ≤ 0.05 against a control group,

b significant at p ≤ 0.05 against the infected group.

**Table 4.** Heavy metals in the muscle of the domestic pigeons and its parasite

**Heavy metals**	**Non-infected pigeon**	**Infected pigeon**	**Parasite**
Fe	2.222 ± 0.02	0.873 ± 0.02 ^a^	1.463 ± 0.02 ^ab^
Cu	0.582 ± 0.02	0.128 ± 0.01 ^a^	0.313 ± 0.01 ^ab^
Zn	0.974 ± 0.02	0.324 ± 0.02 ^a^	0.522 ± 0.02 ^ab^
Cd	0.050 ± 0.001	0.016 ± 0.001 ^a^	0.036 ± 0.001 ^ab^
Cr	0.592 ± 0.02	0.092 ± 0.001 ^a^	0.518 ± 0.02 ^ab^
Co	0.473 ± 0.01	0.085 ± 0.01 ^a^	0.114 ± 0.01 ^ab^

Values are means ±SD.

a significance at p ≤ 0.05 against a control group,

b significant at p ≤ 0.05 against the infected group.

**Table 5.** Heavy metals in the gizzard of the domestic pigeons and its parasite

**Heavy metals**	**Non-infected pigeon**	**Infected pigeon**	**Parasite**
Fe	2.176 ± 0.02	0.741 ± 0.02 ^a^	1.463 ± 0.02 ^ab^
Cu	0.690 ± 0.01	0.120 ± 0.01 ^a^	0.313 ± 0.01 ^ab^
Zn	0.911 ± 0.02	0.409 ± 0.01 ^a^	0.522 ± 0.02 ^ab^
Cd	0.034 ± 0.001	0.010 ± 0.001 ^a^	0.036 ± 0.001 ^ab^
Cr	0.559 ± 0.01	0.267 ± 0.01 ^a^	0.518 ± 0.02 ^ab^
Co	0.166 ± 0.01	0.086 ± 0.001 ^a^	0.114 ± 0.01 ^ab^

Values are means ±SD.

a significance at p ≤ 0.05 against a control group,

b significant at p ≤ 0.05 against the infected group.

Parasites were also capable of accumulating higher levels of metals than those in the tissues of infected pigeons. The bioaccumulation factor was more significant in muscles than in other tissues of infected pigeons with *H. truncata*/*C. livia* model (Table 6). The highest BF registered by *H. truncata* for Cr was 5.630 times that in the host muscles. On the other hand, this model revealed that the lowest BF for Co was 1.096 times that in the host liver.

**Table 6.** Bioaccumulation of heavy metals concerning nematode parasite/pigeon model

**Heavy metals**	**C_*[Hadjelia truncata]*_/C_*[pigeon organ]*_**		

	**Liver**	**Muscles**	**Gizzard**
Fe	1.431	1.675	1.974
Cu	2.371	2.445	2.608
Zn	2.063	1.611	1.276
Cd	3.272	2.250	3.600
Cr	1.519	5.630	1.940
Co	1.096	1.341	1.325

## Discussion

Domestic pigeons play an essential role in the social economy throughout the world (Aldamigh et al., 2022). Endoparasites, especially gastrointestinal nematodes, are responsible for severe health problems in domestic pigeons resulting in severe economic losses (Adang et al., 2008). This study explored the *Hadjelia* species that were present in domestic pigeons during the observation period, their association with histopathological changes, and their role as biological indicators for environmental pollution.

In this study, 29.16% (14/48) of *C. livia* specimens were found to be naturally infected with the adult *Hadjelia* species. Few studies in other geographical regions of the world have reported the prevalence of this parasite. This rate was quite similar to the rate of infection, 27.27%, that was recorded by Shubber (2010) on *C. livia* collected from Al-Diwamiya Province, Central Iraq. On the contrary, however, Ibrahim et al. (1995), Al-Moussawi (2008) and Radfar et al. (2012) reported low prevalence rates of 3.1%, 15.9%, and 17.39% for the domestic pigeons in Egypt, Iraq and Iran infected with the same parasites, respectively. The recovered *H. truncate* species has been identified as an avian parasite and found in the gizzard of some bird species.

This study represents the first report determining the prevalence of this parasite in Riyadh, Saudi Arabia. Previously, few studies have reported pigeon infection with this parasite in Egypt (Tadros and Iskander, 1975; Ibrahim et al., 1995; Abd-Alhadi and Al-Awady, 2020), Iraq (Al-Attar and Abdul-Aziz, 1985; Shubber, 2010; Mohammad and Al-Moussawi, 2011; Al-Moussawi, 2015; Al-Moussawi and Jassim, 2015; Jameel et al., 2016), Cyprus (Appleby et al., 1995), Iran (Razmi et al., 2007; Radfar et al., 2012; Nabavi et al., 2013; Khordadmehr et al., 2018), and California (Sentíes-Cué et al., 2011; Naem et al., 2013).

The morphological characteristics of the habronematid species recovered from the gizzard of pigeons were consistent with the key descriptions previously presented for the *Hadjelia* genus, especially *H. truncate* Cram (1927), Baer (1954), Chabaud (1975), Tadros and Iskander (1975), Appleby et al. (1995), and Junker and Boomker (2007). A comparison of our data with the morphologic measurements made in the previous studies, especially by Al-Moussawi (2015), revealed similar measurements in the main morphological features for the adult parasite stages, particularly in the length of the worms and/or the spicules, the presence of two large trilobed lips, the characteristic caudal region for males of spirurid-type with unequal spicules and number/distribution of papillae, and the position/appearance of the vulva for females. The hypothesis for the presence of these adult stages is consistent with that of Yazwinski and Tucker (2008), who reported the susceptibility of pigeons to infection after ingesting the intermediate host, possibly an arthropod. The pigeons then harbored the infective stage until it was released in the ventriculus lumen and burrowed into the koilin layer to develop into the adult stage. Therefore, it could be identified as *H. truncata* on both morphological and morphometric levels.

Analysis of the 18S rDNA sequences obtained in the present study showed two haplotypes with only one transition at position 116 of the alignment. Sequences obtained from the present study grouped with Habronematidae confirming the morphological identity of the worm detected in the present study. Kelly et al. (2013) obtained partial 18S rDNA sequences from *H. truncata* from California, which were identical, and the representative sequence they submitted was found to be identical to the sequence OR122274 that was obtained in the present study.

One of the partial 18S rDNA sequences of *H. truncata* from the same region obtained from Iraq was highly variable compared to the sequences obtained in the present study, being variable at 14 sites. There was no sequence of *H. truncata* related to the barcoding gene (COX I) in GenBank, and our sequences (OR122277 to OR122279) constitute the first and the only sequences for the pigeon nematode (*H. truncata*) available for comparison with related taxa. Three haplotypes of COX I DNA sequences were recognized in the present study, and there was no change in the amino-acid composition of the translated sequences except for one (OR12227), where serine (S) appeared where phenylalanine (F) had been on the other two sequences (OR122278 and OR122279).

The current study confirms the harmful effects on the host pigeon from nematode parasites that have been reported previously (Appleby et al., 1995). The most prominent pathological findings in the gizzards of the infected *C. livia that* were observed included the enlarged gizzard, as well as distortion and necrosis in the whole structural design and architectural disintegration in the affected tissue. These findings agreed with those in previous studies by Appleby et al. (1995), Audu et al. (2004), Razmi et al. (2007), and Sentíes-Cué et al. (2011), who reported histopathological changes underneath the koilin layer of pigeons infected with *H. truncata*. Ibrahim et al. (1995) attributed the enlargement of the gizzard to the thickening of the cuticle and connective tissue proliferation in the muscular layer. The degenerative changes and necrosis observed in the epithelial layer could also be induced by the local effect of the parasite and/or its toxic products. Alborzi and Rahbar (2012) indicated that the intensity of worms reflects the degree of histological damage.

One of the biggest issues facing the earth today is environmental pollution. To identify pollutants in specific contaminants, biological indicators such as microorganisms are exploited (Zaghloul et al., 2020; Baghdadi et al., 2023). Our study displays the extent of metal accumulation in domestic pigeons and their parasites. Our findings supported the previous study of Al Quraishy et al. (2019), which found that accumulation of heavy metals in various organs was higher in non-infected pigeons than in infected ones. Our results regarding heavy metal accumulation in pigeon tissue also corroborate the findings of Ghita et al. (2009), who reported high concentrations of trace elements in avian feed, which not only accumulated in various body tissues but also polluted the soil and water on the avian litter.

In addition, Boncompagni et al. (2003) reported that higher concentrations of these metals may affect metabolic processes through the replacement of essential elements at the active sites of biologically important molecules, thus indirectly inducing nutritional deficiencies. Our study found significantly higher concentrations of six metals (Fe, Cu, Zn, Cd, Cr, and Co) in parasitic species *H. truncata* than in host pigeon tissues. This finding is corroborated by Taraschewski (2000), Sures et al. (2002), Barus et al. (2003), Thielen et al. (2004), Malek et al. (2007), and Abdel-Ghaffar et al. (2015). Leite et al. (2017) suggested that the bioaccumulation of helminth parasites might reflect a higher capacity in the host to excrete heavy metals, and could be considered advantageous to the parasites, which could act as heavy metal sanitizers for the host. This study showed lower BF for Co in the liver, which agreed with the finding in Sures and Roy (2001) that high metal levels in the host tissue and a correspondingly lower BF indicate longer exposure.

## Conclusion

The present study provides valuable information about the occurrence of a habronematid species identified as *Hadjelia truncata* and its negative impact on domestic pigeons. In addition, we have provided a complete description of this parasitic species at both the morphological and the morphometric levels. This study also demonstrates that 18S rDNA and COX I gene regions yield a unique sequence and confirm its taxonomic position within Habronematidae. This pigeon Nematoda appears to be a suitable bioindicator for metals in the environment. Further investigations into *Hadjelia* species should investigate more of its genes.
